# Trends in Total and Low-Density Lipoprotein Cholesterol among U.S. Adults: Contributions of Changes in Dietary Fat Intake and Use of Cholesterol-Lowering Medications

**DOI:** 10.1371/journal.pone.0065228

**Published:** 2013-05-22

**Authors:** Earl S. Ford, Simon Capewell

**Affiliations:** 1 Division of Population Health, National Center for Chronic Disease Prevention and Health Promotion, Centers for Disease Control and Prevention, Atlanta, Georgia, United States; 2 Department of Public Health, University of Liverpool, Liverpool, United Kingdom; Brigham & Women's Hospital, and Harvard Medical School, United States of America

## Abstract

**Objective:**

Our aim was to examine the relative contributions of changes in dietary fat intake and use of cholesterol-lowering medications to changes in concentrations of total cholesterol among adults in the United States from 1988–1994 to 2007–2008.

**Method:**

We used data from adults aged 20–74 years who participated in National Health and Nutrition Examination Surveys from 1988–1994 to 2007–2008. The effect of change in dietary fat intake on concentrations of total cholesterol was estimated by the use of equations developed by Keys, Hegsted, and successors.

**Results:**

Age-adjusted mean concentrations of total cholesterol were 5.60 mmol/L (216 mg/dl) during 1988–1994 falling to 5.09 mmol/L (197 mg/dl) in 2007–2008 (*P*<0.001). No significant changes in the intake of total fat, saturated fat, polyunsaturated fat, and dietary cholesterol were observed from 1988–1994 to 2007–2008. However, the age-adjusted use of cholesterol-lowering medications increased from 1.6% to 12.5% (*P*<0.001). The various equations suggested that changes in dietary fat made minimal contributions to the observed trend in mean concentrations of total cholesterol. The increased use of cholesterol-lowering medications was estimated to account for approximately 46% of the change.

**Discussion:**

Mean concentrations of total cholesterol among adults in the United States have declined by ∼4% since 1988–1994. The increased use of cholesterol-lowering medications has apparently accounted for about half of this small fall. Further substantial decreases in cholesterol might be potentially achievable by implementing effective and feasible public health interventions to promote the consumption of a more healthful diet by US adults.

**Disclaimer:**

The findings and conclusions in this article are those of the authors and do not necessarily represent the official position of the Centers for Disease Control and Prevention.

## Introduction

The intake of dietary fats is an important lifestyle factor that affects concentrations of total cholesterol. Work by Keys and by Hegsted and their colleagues resulted in the development of equations that related changes in the intake of saturated fatty acids, polyunsaturated fatty acids, and dietary cholesterol to changes in concentrations of total cholesterol [1], [2]. The discovery and subsequent development of statins (HMG-CoA reductase inhibitors or 3-hydroxy-3-methylglutaryl CoA reductase inhibitors) provided clinicians with a powerful new class of medications to improve cholesterol concentrations in their patients.

Understanding to what extent dietary change and use of cholesterol-lowering medications affected the trend in the concentrations of total cholesterol in the U.S. population is important firstly to yield insights into the dynamics of the trend in the mean concentration of total cholesterol and secondly to inform future public health and clinical approaches necessary to achieve the Healthy People 2020 objective that calls for reducing the mean concentration of total cholesterol to 4.60 mmol/L (177.9 mg/dl) [3]. The principal objective of this study was to estimate the impact of changes in dietary fat intake and use of cholesterol-lowering medications on concentrations of total cholesterol subsequent to 1987 when the first statin was introduced in the United States. In addition, secondary objectives included 1) examining trends in dietary fat intake among U.S. adults and 2) examining trends in the use of cholesterol-lowering medications among U.S. adults. Although the time frame for the study covers the period from 1988–1994 to 2007–2008, we also present data concerning trends in dietary fat intake among U.S. adults starting with the period 1971–1975 to give a historical perspective.

## Materials and Methods

### Ethics statement

Because our study used existing public-use data sets that are readily available, our study was exempt from human subjects review.

### Data sources

We used data from eight NHANES cycles: 1971–1975, 1976–1980, 1988–1994, 1999–2000, 2001–2002, 2003–2004, 2005–2006, and 2007–2008. In brief, each survey employed a complex multistage cluster design to select samples of the civilian, noninstitutionalized population in the United States. Selected participants were interviewed in their homes and invited for an examination that included completing additional questionnaires, receiving various examinations, and providing biological specimens including blood and urine. Detailed descriptions of each of these surveys may be found elsewhere [4]–[9].

### Variables

The methods used to measure serum total cholesterol and low-density lipoprotein cholesterol have been summarized elsewhere [10], [11].

Dietary data were obtained by using a single 24-hour recall from 1971–1975 through 2001–2002 and two 24-hour recalls starting with the 2003–2004 survey [12], [13]. To maintain consistency across surveys, we used only data from the first 24-hour recall for surveys that collected more than one. Data files of all surveys contained information for the intake of total fat, saturated fat, dietary cholesterol, and total energy. Starting with NHANES III (1988–1994), data files also contained information for the intake of polyunsaturated fat and monounsaturated fat.

We used the following formula by Keys and colleagues to estimate the effect of changes in the intake of saturated fat, polyunsaturated fat, and dietary cholesterol on serum concentrations of total cholesterol (mg/dl): 1.35 * (2ΔS – ΔP)+1.5 * ΔC^0.5^, where S = % of energy from saturated fatty acids, P = % of energy from polyunsaturated fatty acids, and C = dietary cholesterol [mg/1000 kcal] [1]. We also used a formula developed by Howell and colleagues to estimate the effect of changes in the three dietary sources of fat on concentrations of total cholesterol (mg/dl): 1.918 * ΔS−0.900 * ΔP+0.0222 * ΔC [14]. S and P are both expressed as percent of total energy, whereas C is expressed in mg/day. In addition, we estimated the change in fasting concentrations of low-density lipoprotein cholesterol (mmol/L) from the equation: 0.036 ΔS – 0.022 ΔP – 0.008 ΔM+0.0005 * ΔC, where S = % of energy from saturated fatty acids, P = % of energy from polyunsaturated fatty acids, M = % of energy from monounsaturated fatty acids, and C = dietary cholesterol [mg/day] [15].

Participants could report up to 16 prescription medications in NHANES III and up to 20 prescription medications in NHANES 2007–2008 and, if available, were asked to show them to the interviewers. Participants who had been prescribed one of the following medications were considered to be using cholesterol-lowering medications: atorvastatin, cerivastatin, fluvastatin, lovastatin, lovastatin & niacin, pravastatin, simvastatin, ezetimibe & simvastatin, rosuvastatin, cholestyramine, colestipol, colesevelam, and ezetimibe.

To estimate the possible effect of the use of these medications on the decrease in mean concentrations of total cholesterol and low-density lipoprotein cholesterol, we used the following formula: Δ prevalence of cholesterol-lowering medications use from 1988–1994 to 2007–2008 * estimated reduction in total cholesterol caused by these medications * suboptimal dosing * compliance/change in mean concentrations of lipid.

We estimated the proportion of suboptimal dosing at 75% [16], [17]. Several studies peg compliance with the use of statins at 50% to 55% [18]–[20], and we used a value of 55%.

We also estimated predicted mean concentrations of total cholesterol in the absence of use of cholesterol-lowering medications for 1988–1994 and 2007–2008. For these calculations, we assumed that cholesterol-lowering medications lower concentration of total cholesterol by 2.0 mmol/L (77 mg/dl).

### Data analysis

We limited our analysis to adults aged 20–74 years. We chose 74 years as our upper age limit as that age represented the upper age limit in early surveys such as NHANES I. The distribution of the projected 2000 US population was used to perform age-adjustment using the direct method [21]. Using time in years as determined from the approximate midpoints of the surveys, tests for trend adjusted for age as a continuous covariate were calculated using linear regression for continuous variables and log-linear regression for dichotomous variables. Analyses that incorporated sampling weights and design variables were conducted using SUDAAN to account for the complex sampling designs of the surveys. Sample sizes are shown as unweighted numbers.

## Results

The numbers of adults aged 20–74 years who attended the NHANES examinations were 16165 in 1971–1975, 15364 in 1976–1980, 16115 in 1988–1994, 4201 in 1999–2000, 4616 in 2001–2002, 4251 in 2003–2004, 4381 in 2005–2006, and 5168 in NHANES 2007–2008. Sample sizes for the various analyses were generally less due to missing values for the study variables. These sample sizes pertain to the descriptive epidemiologic analyses and not to the modeling part of the study that employed group means from two time periods.

The mean age was 42.8 years in NHANES I and 44.0 years in NHANES 2007–2008 (*P* for linear trend <0.001). The percentage of participants who were men was about 47% in NHANES I and 48% in NHANES 2007–2008 (*P* for linear trend = 0.140). The percentage of non-Hispanic white participants decreased from 89% to 69% (*P* for linear trend <0.001). The percentage of participants who had graduated with at least a high school education increased from 64% to 81% (*P* for linear trend <0.001).

### Total cholesterol and low-density lipoprotein cholesterol

**Figure 1:**
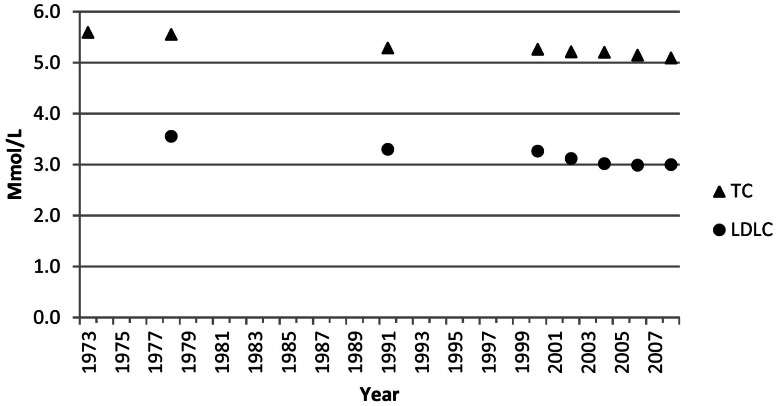
Age-Adjusted Mean Concentrations of Total Cholesterol (TC) and Low-Density Lipoprotein Cholesterol (LDLC) among U.S. Adults Aged 20–74 Years. Data points were plotted at the approximate midpoint of the study period.

**Figure 2:**
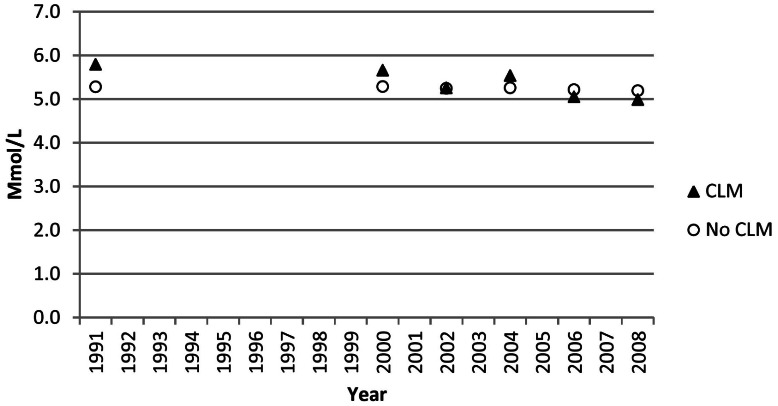
Age-Adjusted Mean Concentrations of Total Cholesterol among U.S. Adults Aged 20–74 Years, by use of cholesterol-lowering medications (CLM). Data points were plotted at the approximate midpoint of the study period.

The age-adjusted mean concentration of total cholesterol decreased from 5.60 mmol/L (216 mg/dl) during 1971–1975 to 5.09 mmol/L (197 mg/dl) during NHANES 2007–2008 (*P*<0.001) (**Figure 1**). The change was particularly pronounced among users of cholesterol-lowering medications among whom mean concentrations of total cholesterol decreased from 5.80 mmol/L (224 mg/dl) to 4.99 mmol/L (193 mg/dl) (P<0.001) whereas concentrations decreased from 5.29 mmol/L (204 mg/dl) to 5.19 mmol/L (201 mg/dl) (P = 0.022) among adults who did not use cholesterol-lowering medications (**Figure 2**). The age-adjusted mean concentrations of low-density lipoprotein cholesterol were 3.55 mmol/L (137 mg/dl) during 1976–1980 and 3.00 (116 mg/dl) during NHANES 2007–2008 (*P*<0.001). For both total cholesterol and low-density lipoprotein cholesterol, tests of interaction indicated that declines in concentration of these lipids were stronger among participants who used cholesterol-lowering medications than among those who did not (p interaction <0.001 for both lipids).

### Dietary fat intake from 1971–1975 to 2007–2008

The absolute age-adjusted mean intake of total fat increased significantly (*P* = 0.003) (**Table 1**). The largest mean was recorded for the 2003–2004 period. When expressed as a percentage of energy intake, total fat intake decreased significantly largely driven by the decrease from 1976–1980 to 1999–2000 (*P*<0.001). The different impressions regarding the direction of change for mean intake of total fat intake and total fat intake expressed as a percentage of energy intake reflects the increase in energy intake during this period. The mean intake of saturated fat, both in absolute terms (*P* = 0.002) and expressed as a percentage of energy intake (*P*<0.001), decreased significantly reflecting the large decline from 1971–1975 to 2001–2002. Dietary cholesterol decreased significantly, both in absolute terms and expressed per 1,000 kcals, largely reflecting a drop of about 27% from 1971–1975 to 1988–1994. Dietary cholesterol intakes then rose slightly between 1988–1994 and 2007–2008. When we used the data for two 24-hour recalls for the 2-year cycles of 2003–2004, 2005–2006, and 2007–2008, the results changed minimally.

**Table 1:** Age-Adjusted Mean Intakes of Fats and Energy among U.S. Adults Aged 20–74 Years.

		Saturated fat		Polyunsaturated fat		Monounsaturated fat		Total fat		Cholesterol		Total energy intake
	N	g/day	% kcal	g/day	% kcal	g/day	% kcal	g/day	% kcal	mg/day	mg/1000 Kcal/day	Kcal/day
NHANES I (1971–1975)	13050	29.6 (0.4)	13.2 (0.1)	—	—	—	—	81.4 (0.9)	36.4 (0.1)	395.3 (5.6)	209.9 (2.4)	1969.9 (18.9)
NHANES II (1976–1980)	11864	28.9 (0.4)	12.8 (0.1)	—	—	—	—	80.7 (0.9)	36.3 (0.2)	353.6 (5.2)	186.9 (1.8)	1959.9 (16.3)
NHANES III (1988–1994)	14167	28.5 (0.4)	11.3 (0.1)	18.1 (0.3)	7.2 (0.1)	32.1 (0.4)	12.6 (0.1)	85.1 (1.1)	33.6 (0.2)	288.5 (5.1)	130.6 (1.8)	2215.7 (17.5)
NHANES 1999–2000	3735	27.6 (0.4)	11.0 (0.2)	17.0 (0.3)	6.9 (0.1)	31.3 (0.4)	12.4 (0.2)	82.2 (1.1)	32.9 (0.4)	290.6 (4.7)	135.2 (2.4)	2216.7 (31.5)
NHANES 2001–2002	4173	27.1 (0.4)	10.6 (0.1)	17.0 (0.3)	6.8 (0.1)	30.9 (0.4)	12.1 (0.1)	84.0 (1.0)	33.2 (0.2)	288.5 (3.7)	129.7 (1.7)	2248.4 (25.8)
NHANES 2003–2004	3790	28.4 (0.4)	11.1 (0.1)	18.1 (0.3)	7.2 (0.1)	32.5 (0.4)	12.6 (0.1)	86.2 (1.0)	33.8 (0.3)	295.0 (6.6)	134.2 (2.3)	2274.7 (17.3)
NHANES 2005–2006	4021	28.8 (0.5)	11.3 (0.1)	18.0 (0.4)	7.2 (0.1)	31.7 (0.6)	12.4 (0.1)	85.9 (1.6)	33.8 (0.3)	300.3 (4.5)	134.7 (1.6)	2251.3 (29.5)
NHANES 2007–2008	4761	27.3 (0.6)	11.1 (0.1)	17.5 (0.3)	7.2 (0.1)	30.4 (0.6)	12.3 (0.1)	82.4 (1.6)	33.6 (0.2)	298.2 (7.0)	137.5 (2.4)	2165.1 (31.1)
P for linear trend from 1971–1975 to 2007–2008		0.002	<0.001	—	—	—	—	0.003	<0.001	<0.001	<0.001	<0.001
P for linear trend from 1988–1994 to 2007–2008		0.576	0.669	0.812	0.330	0.137	0.090	0.782	0.516	0.132	0.039	0.890

### Cholesterol-lowering medications from 1988–1994 to 2007–2008

**Figure 3:**
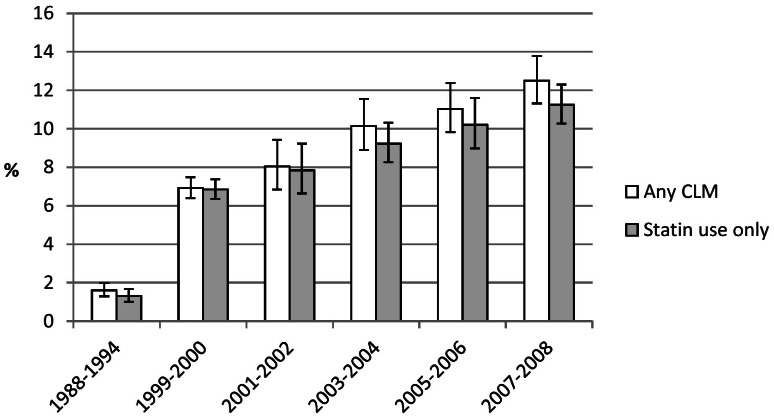
Age-Adjusted Percent Use (95% Confidence Interval) of Cholesterol-Lowering Medications (CLM) and Statins among U.S. Adults Aged 20–74 Years.

The use of any cholesterol-lowering medications increased progressively from 1.6% during 1988–1994 to 12.5% 2007–2008 (*P*<0.001) (**Figure 3**). The vast majority of this change was attributable to the strong increase in the use of statins.

### Effect of changes in dietary fat intake on concentrations of total cholesterol and low-density lipoprotein cholesterol from 1988–1994 to 2007–2008

**Figure 4:**
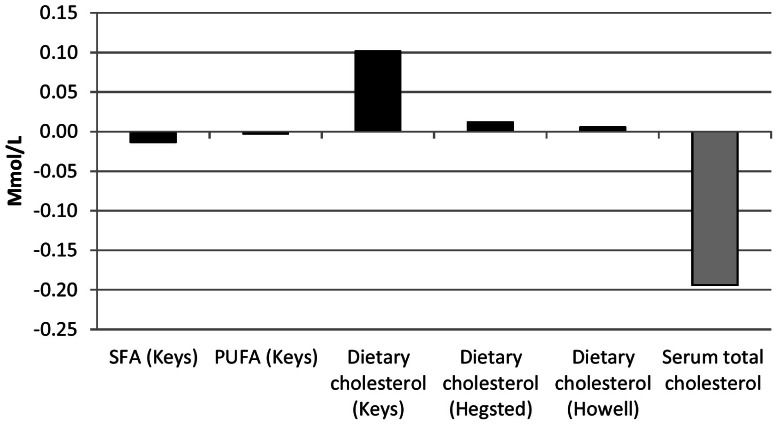
Predicted Changes in Concentration of Total Cholesterol Attributable to Changes in the Dietary Intake of Saturated Fat (SFA), Polyunsaturated Fat (PUFA), and Dietary Cholesterol and Actual Change in Concentration of Total Cholesterol (Grey Bar) among U.S. Adults Aged 20–74 Years from 1988–1994 to 2007–2008.

The changes in the intake of saturated fat and polyunsaturated fat had a negligible effect on concentrations of total cholesterol (**Figure 4**). The Keys equation suggested that the increase in the intake of dietary cholesterol after 1988–1994 should have increased mean concentrations of total cholesterol by about 0.10 mmol/L (3.9 mg/dl). Thus, the net effect of the changes in fat intake should have increased mean concentrations of total cholesterol by about 0.09 mmol/L (3.3 mg/dl). Yet, the mean age-adjusted concentration of total cholesterol actually decreased by about 0.19 mmol/L (7.5 mg/dl). Estimating mean intakes of dietary fats with two 24-hour dietary recalls for the periods 2003–2004, 2005–2006, and 2007–2008 had very little effect on analyses.

A subsequent analysis by Hegsted suggested that earlier studies had overestimated the relationship between changes in the intake of dietary cholesterol and changes in circulating concentrations of total cholesterol [22]. Using a revised equation he and colleagues proposed [23], concentrations of total cholesterol were predicted to have increased by approximately 0.02 mmol/L (0.5 mg/dl) (**Figure 4**). Using this value, the net predicted change in total cholesterol attributable to changes in the three dietary sources of fats was −0.0006 mmol/L (0.02 mg/dl).

When we applied the formula developed by Howell and colleagues to our data [14], changes in the intake of saturated fat and polyunsaturated fat would have decreased concentrations of total cholesterol by 0.01 mmol/L (0.4 mg/dl) and 0.002 mmol/L (0.1 mg/dl), respectively. Changes in the intake of dietary cholesterol would have increased concentrations of total cholesterol by 0.01 mmol/L (0.2 mg/dl) (**Figure 4**). Thus, the net effect of these changes would have decreased concentrations of total cholesterol by 0.01 mmol/L (0.2 mg/dl).

The changes in the intake of dietary fatty acids and cholesterol also produced essentially no change in the concentration of low-density lipoprotein cholesterol (data not shown).

### Effect of changes in the use of cholesterol-lowering medications on mean concentrations of total cholesterol and low-density lipoprotein cholesterol

**Figure 5:**
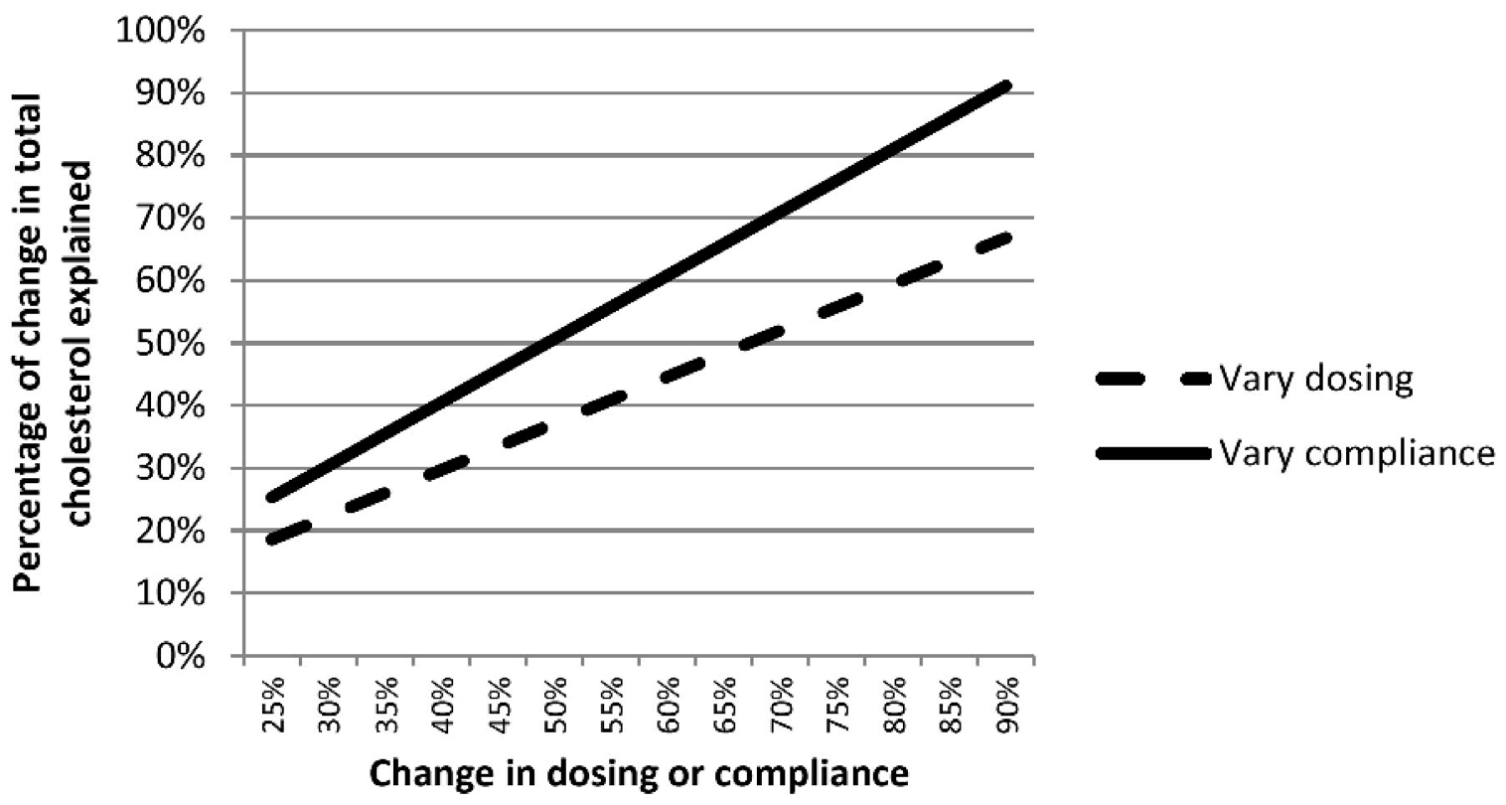
Sensitivity Analysis Illustrating Potential Effects of Varying Assumptions Concerning the Levels of Suboptimal Dosing and Compliance on the Percentage of the Decline in Concentrations of Total Cholesterol Explained by the Use of Cholesterol-Lowering Medications among U.S. Adults Aged 20–74 years from 1988–1994 to 2007–2008.

**Figure 6:**
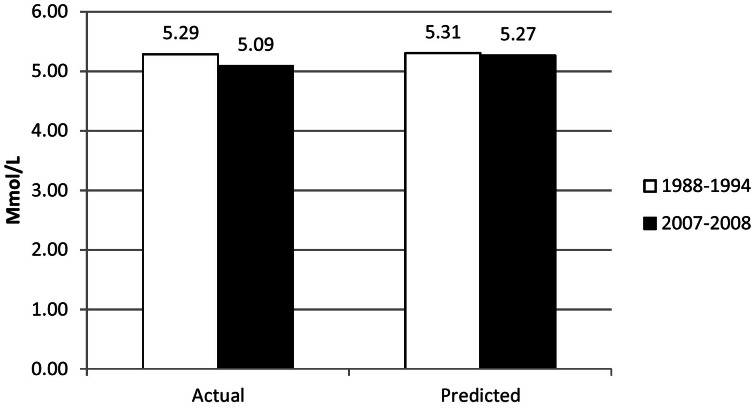
Age-Adjusted Actual and Predicted Mean Concentrations of Total Cholesterol among U.S. Adults Aged 20–74 Years.

The increased use of cholesterol-lowering medications resulted in decreases of 0.09 mmol/L (3.5 mg/dl) of total cholesterol and 0.09 mmol/L (3.5 mg/dl) of low-density lipoprotein cholesterol, thus representing approximately 46% of the decrease in mean concentrations of total cholesterol and 29% of the decrease in low-density lipoprotein cholesterol. The results of sensitivity analyses whereby the levels of suboptimal dosing and the levels of compliance were allowed to vary from 25% to 90% are shown in **Figure 5**. Alternatively, we estimated that in the absence of the use of cholesterol-lowering medications, mean concentrations of total cholesterol would have been 5.31 mmol/L (205 mg/dl) during 1988–1994 and 5.27 mmol/L (204 mg/dl) during 2007–2008 (**Figure 6**). These recalculated means assumed that cholesterol-lowering medications would have lowered concentrations of total cholesterol by about 2.0 mmol/L (77 mg/dl) and that compliance was 55%.

## Discussion

Our analyses suggest that the modest recent declines in the mean concentration of total cholesterol since 1988–1994 mainly reflect the increased use of cholesterol-lowering medications. In contrast, changes in the intake of various dietary fats had little effect on concentrations of total cholesterol. Because we used data from NHANES that are representative of the noninstitutionalized civilian population, our results are largely reflective of the U.S. population aged 20–74 years.

The role of cholesterol-lowering medications in explaining almost half of the small decrease in mean concentration of total cholesterol among U.S. adults during the past two decades contrasts markedly with findings from Finland and Sweden [24], [25]. Prior to the introduction of statins, mean concentrations of total cholesterol in Finland and Sweden were higher than those in the United States [26], [27]. Furthermore, mean intake of saturated fat was considerably higher in Finland and likely in Sweden than in the United States [25], [28]. During this period, dietary modification was the principal option to bring about population-wide reductions in mean concentrations of total cholesterol. As a result of the North Karelia project, a paradigm to change dietary intake on a population-wide basis in Finland was developed [29]. Thus a focus on modifying the intake of dietary fats in Finland and Sweden produced a pronounced impact of dietary efforts on trends in lipid concentrations. In contrast, higher use of cholesterol-lowering medications in the United States than Finland and Sweden at approximate similar points in time helps to explain the differences between the countries in the relative importance of the two major contributing factors in lowering mean concentrations of total cholesterol [24], [25].

As acknowledged by the 2010 US Dietary Guidelines [30] and as shown by our analyses, the intakes of total fat, saturated fat, polyunsaturated fat have changed little in the past two decades, and thus appear to have contributed little to lowering population mean concentrations of total cholesterol and low-density lipoprotein cholesterol in the United States. Furthermore, the intake of dietary cholesterol has inched upwards since 1988–1994.

In contrast, the use of cholesterol-lowering medications, mainly statins, has progressively increased during the past two decades and currently appears to be rising by about one percentage point per year among U.S. adults. Given the more or less stationary patterns in the consumption of dietary fats, future progress in lowering mean concentrations of total cholesterol in the United States will likely be heavily dependent on the increased use of these medications. In contrast, dietary interventions to decrease concentrations of total cholesterol are potentially powerful but currently relatively neglected in the United States. Furthermore, increased pressures to eliminate industrial trans fats and decrease the content of saturated fats in the food supply may reduce total cholesterol concentrations in the U.S. population in future.

Several study limitations should be acknowledged. The study used an ecologic design that is subject to various methodologic issues [31]. Because we used data from national surveys that included different sets of participants, we were unable to work with data at the individual level and instead worked with grouped data. Thus, the results of our study are potentially subject to an ecologic bias.

Dietary data were collected with a single 24-hour recall in most surveys, and when two such recalls were obtained, we used only the first one to maintain consistency across surveys. Although a larger number of recalls would have yielded better estimates of intakes, mean intakes of such recalls are generally thought to provide reasonable estimates of actual population means. Also, food data bases used to calculate dietary intake have changed, and the operational details of the 24-hour recall used in NHANES have gradually evolved.

Our estimates explained almost half of the modest fall in concentrations in total cholesterol and about one-fourth of the fall in low-density lipoprotein cholesterol. This gap may be due to imprecise estimates of suboptimal dosing and compliance with the use of cholesterol-lowering medications, the contribution of dietary components other than dietary fats, or other factors that affect concentrations of total cholesterol.

Although data on the intake of trans-fatty acids were not available, a recent analysis showed that concentrations of these fats in white U.S. adults halved from 2000 to 2009 suggesting that the intake of these fats decreased [32]. Thus, some of the unexplained proportion of the decrease concentrations of total and low-density lipoprotein cholesterol may reflect decreased consumption of trans-fatty acids. Replacing 1% of transfats with a mix of mono and poly-unsaturated fats should reduce total serum cholesterol by about 0.04 mmol/L (1.5 mg/dl) [33].

After accounting for suboptimal dosing and compliance, we estimated that the reduction in concentrations of total cholesterol by cholesterol-lowering medications amounted to 0.825 mmol/L (32 mg/dl). Unfortunately, good national data about these parameters are unavailable, and we relied on less representative data. Other authors have used different estimates and assumed that the use of cholesterol-lowering medications decreased concentrations of total cholesterol by 25% or by 1.5 mmol/L (58 mg/dl) [24], [25]. Had we used the more optimistic estimate of 1.5 mmol/L (58 mg/dl) instead of 0.825 mmol/L (32 mg/dl), the increased use of cholesterol-lowering medications would have accounted for 82% of the decrease in mean concentrations of total cholesterol and 50% of the decrease in mean concentrations of low-density lipoprotein cholesterol.

Because estimates of low-density lipoprotein cholesterol were calculated using the Friedewald equation [34], measurements of concentrations of high-density lipoprotein cholesterol and triglycerides are critical. Mean concentrations of high-density lipoprotein cholesterol increased among U.S. adults during the study period, and changes in the methods used to measure high-density lipoprotein cholesterol were proposed as one explanation for the apparent increase [11]. If this explanation holds, the change in the concentration of low-density lipoprotein cholesterol would have been overestimated and the change attributed to increased use of cholesterol-lowering medications underestimated.

In conclusion, our results suggest that the modest reductions in mean concentrations of total cholesterol in the U.S. population during the past two decades largely reflect the increased use of cholesterol-lowering medications rather than a decreased dietary intake of fats. The experience from other countries [24], [25] and from the United States [35], [36] prior to the rapid escalation of the use of cholesterol-lowering medications emphasizes that dietary changes could still play a powerful role in driving down the mean concentration of total cholesterol in the population. Our analyses may contribute to the dialogue about the need for effective public health policy to improve lipid concentrations through better diet in the United States particularly in light of the new Million Heart Initiative that seeks to prevent one million heart attacks over the next five years in part through public health efforts to improve diet [37]. Failing that, escalating expenditure on cholesterol-lowering medications will likely continue to be the principal determinant of trends in total cholesterol concentrations in the United States.
